# Analysis of interception problems in donning and doffing personal protective equipment in a large cabin hospital during the COVID-19 pandemic: a real world study

**DOI:** 10.1038/s41598-026-39259-z

**Published:** 2026-02-07

**Authors:** Zhanjie Li, Chuanyuan Tang, Feng Zang, Xinyue Zhang, Jun Wu, Weihong Zhang, Chuanlong Zhu

**Affiliations:** 1 Department of Infection Control, The First Affiliated Hospital with Nanjing Medical University, 300 Guangzhou Road, Nanjing, 210029 Jiangsu China; 2 Intensive Care Unit, The First Affiliated Hospital with Nanjing Medical University, Nanjing, 210029 Jiangsu China; 3 Department of Hepatobiliary Center, The First Affiliated Hospital with Nanjing Medical University, Nanjing, 210029 Jiangsu China; 4 Department of Geriatric Cardiology, The First Affiliated Hospital with Nanjing Medical University, Nanjing, 210029 Jiangsu China; 5 Department of Infections Disease, The First Affiliated Hospital with Nanjing Medical University, No.300 Guang Zhou Road, Nanjing, 210029 Jiangsu China

**Keywords:** Large cabin hospital, Donning and doffing of the protective equipment, Problem distribution, Real-world study, Health occupations, Occupational health

## Abstract

Although proper donning and doffing of personal protective equipment (PPE) is critical for infection prevention, detailed characterization of problems encountered during these processes in real-world settings during large-scale infectious disease outbreaks remains insufficient. This study aimed to analyze the characteristics and distribution of interception problems in the process of donning and doffing process in a large cabin hospital during the 2022 COVID-19 pandemic in Shanghai. A prospective, real-world study was designed to collect and analyze data on irregularities observed during personal protective equipment (PPE) donning procedures in cabin hospital operations. The proportion of problems encountered during donning PPE was 5.29% (246/4,652), while during doffing PPE, it was 8.44% (382/4,525) (*P* < 0.001). The primary problem during donning PPE was related to problems with protective clothing, followed by problems with respirators. There was no significant difference in problem distribution among different posts (*P* = 0.459). The problems related to protective clothing mainly focused on loose fitting around the head and neck, making them prone to exposure during donning (56.25%, 99/176) and contamination of the inner surface of protective clothing during doffing (46.43%, 91/196). Respirator-related problems included failure of the seal test during donning PPE (61.54%, 24/39) and shifting or loosening of the respirator during PPE doffing (73.68%, 14/19). These findings identify critical gaps in PPE procedures and highlight the need for targeted training to address these issues, thereby reducing the risk of infection among healthcare personnel in mobile cabin hospitals.

**Supplementary Information:**

The online version contains supplementary material available at 10.1038/s41598-026-39259-z.

## Introduction

Since its outbreak at the end of 2019, coronavirus disease 2019 (COVID-19) has rapidly spread to almost all parts of the world^1,2^. The Omicron variant, first discovered in southern Africa in November 2021, has gradually replaced the Delta variant as the global leading strain^3,4^. The Omicron variant has stronger transmission and immune escape abilities compared to the Delta variant^5,6^. Despite its high transmissibility, the Omicron variant is associated with relatively mild clinical symptoms^7,8^. The experience gained from the COVID-19 fight in Wuhan, Hubei Province in 2020 demonstrated that mobile cabin hospitals can provide a large number of sickbeds and appropriate care for non-critical infected patients^9^, representing an important countermeasure to large-scale or rapidly growing public health emergencies. During the Shanghai epidemic from March to June 2022, more than 600,000 cases of Omicron infection were reported^10^. Against this backdrop, many mobile cabin hospitals were built to admit a huge number of patients with mild symptoms or asymptomatic patients. Large mobile cabin hospitals are characterized by expansive areas, plentiful sickbeds, massive medical staff, cleaning and security personnel, and a complex and frequent process of donning and doffing personal protective equipment (PPE).

Correct utilization of PPE can prevent potential infections through the mouth, nose, eyes, hands, and skin, representing a vital measure in reducing the occupational exposure risk for medical personnel^11^. Standardized and effective donning and doffing PPE are critical components in ensuring its proper use. The core of donning PPE in mobile cabin hospital is “comprehensive coverage” and “tight protection without omission”, while the core of doffing PPE is “overcoming hardness by softness” and “leaving the cabin hospital without pollution”. Cabin hospitals are often hastily established or repurposed amidst a surge in COVID-19 cases^12^ Despite receiving uniform training and supervision on donning and doffing PPE^13^, medical personnel may still grapple with stress, anxiety, and other psychological factors during these procedures, particularly in the days leading up to their deployment to the cabin hospital, potentially leading to confusion in the steps of donning and doffing PPE. Moreover, cleaning, security, and maintenance personnel may lack proficiency in these procedures, further exacerbating concerns.

Previous related studies have largely used simulation- or training-based scenarios, often in the context of highly infectious diseases (for example, Ebola or COVID-19), to document errors and contamination events during donning and doffing^14–21^. However, most of this work has been conducted in controlled settings or among relatively small groups of healthcare workers. In contrast, detailed real-world observational data on specific problems occurring during both donning and doffing, particularly across different occupational groups in large mobile cabin hospitals, remain limited.

This study aims to collate and analyze these relevant interception problems, providing targeted guidance for subsequent training and assessment, and serving as a reference for other medical institutions. This analysis is based on the extensive data generated by large mobile cabin hospitals (> 1,000 beds)^22^, which accurately reflect challenges in real-world operations. Specifically, during the intensive response to the COVID-19 pandemic in Shanghai (April to May 2022), our medical team managed a 1,240-bed cabin hospital, overseeing 9,177 donning and doffing procedures and collecting data on 628 intercepted problems.

## Methods

### Study subjects

This prospective observational analyzed interception problems in the process of donning and doffing PPE among staffs engaged in one of the cabins (1,240 sickbeds) at the Shanghai Lingang mobile cabin hospital (13,608 sickbeds in total) from April 13 to May 7, 2022. The study included 4,652 person-times of donning PPE, with 246 interception problems, and 4,525 person-times of doffing PPE with 382 interception problems. The occupational types included medical personnel (doctor, nurse, and infection control personnel) and three-guarantee personnel (cleaning, security, and maintenance personnel). The PPE used consisted of protective clothing, respirators, gloves, protective screens, disposable medical caps, and disposable shoe covers. In addition, some staff members used tape to secure the cuffs and necks of protective clothing. This study was designed in accordance with the Declaration of Helsinki and approved by the ethics committee of The First Affiliated Hospital with Nanjing Medical University (*Ethic number: 2022-SR-315*). According to local ethics, we have applied for exemption from written informed consent.

### Participants and recruitment

All staff members who worked in the selected cabin during the study period and were required to don and doff PPE when entering or leaving the contaminated or semi‑contaminated zones were consecutively included in this study. No sampling procedure was applied. The study population therefore represents the total workforce at risk of exposure in this cabin during routine operation. Inclusion criteria were: (1) employment as medical personnel (physicians, nurses or infection control staff) or three‑guarantee personnel (cleaning, security or maintenance staff) assigned to the study cabin; (2) direct involvement in activities that required entering the contaminated or semi‑contaminated areas; and (3) completion of the standardized PPE training program based on the national expert consensus before starting duty. Exclusion criteria were: (1) temporary or newly deployed staff who had not completed the full PPE training; and (2) shifts with missing or incomplete supervision records that did not allow reliable identification of the donning or doffing process. The interception records analyzed in this prospective study were originally collected as part of the routine infection prevention and control supervision program in the Fangcang cabin hospital. For the purpose of this study, all individual identifiers were removed, and data were aggregated at the level of person‑times of donning and doffing PPE.

### Supervision and guidance

Full-time infection control supervisors, who were on duty for 24 h, were appointed to supervise, guide, and assist staff in donning and doffing PPE. In accordance with the requirements outlined in the Notice on Issuing Guidelines for the Construction and Management of Makeshift Hospitals in Jiangsu Province (for Trial Implementation) (No.32 [2022]), infection control supervisors were provided on a 10:100 basis for medical personnel.

### Data collection process

Standardized record forms, namely the donning supervision form and the doffing supervision form, were developed to facilitate infection control supervisors in documenting each donning and doffing event (Supplemental Tables 1, 2). In this study, “interception problems” were defined as any procedural irregularities or non-compliant behaviors observed during the PPE procedures that were identified and corrected in real-time by supervisors. These represented potential safety breaches that were “intercepted” and rectified before they could lead to actual exposure risk. During each shift, the on‑duty supervisors completed the forms while observing staff members donning or doffing PPE and followed the general guidelines for the process, which required proper fitting and sizing of PPE with no gaps around the neck and facial areas during donning and prevention of cross‑contamination by ensuring that contaminated parts did not come into contact with clean ones during doffing. At the end of each shift, a dedicated mobile phone was used to take photographs of the completed forms, which were then uploaded to the WeChat group of the infection control team. A designated member of the team was responsible for collecting all forms, checking their completeness, and organizing, coding and summarizing the data on a daily basis. The collected data were subsequently used for quantitative analysis of interception problems during donning and doffing of PPE.

### Variables collected

For each supervised donning or doffing of PPE, infection control supervisors completed a standardized supervision form. The following variables were recorded for every event: date and time of the observation, the cabin zone (four-zone layout of the pod), the type of PPE process (donning or doffing), and personnel characteristics including occupation category (doctor, nurse, infection control personnel, cleaning personnel, security personnel, or maintenance personnel) and the original hospital of the staff member. For each donning or doffing event, supervisors documented whether any deviation from the standard procedure occurred and provided a detailed description of the problem (for example, “mask air tightness test not performed”, “inner hat exposed”, or “protective clothing loose at the head and neck, easily exposed”). During donning, problems were categorized as problems related to protective clothing, problems related to respirators, problems related to gloves, or other problems (such as incorrect position of the protective screen, uncovered hair, or exposed inner hat). During doffing, problems were categorized as problems related to respirators, problems related to gloves, or other problems, including issues with undressing order, inadequate hand hygiene, improper disposal of waste, and non-standard removal of the protective screen.

### Quality control measures

The infection control group established a dedicated quality control group for data reporting, consisting of one group leader and one group member. Their tasks included clarifying the recording methods of the supervision form and providing training all the infection control supervisors. The training covered the standard donning and doffing procedures, which were guided by the consensus of Chinese experts^23^, as well as the operational definitions of each type of interception problem and typical examples of correct and incorrect practices. Additionally, the quality control group was responsible for collecting the supervision form uploaded in the WeChat group every day, ensuring proper completion, and promptly providing feedback within the group.

### Classification of interception problems

In this study, a reportable interception problem was defined as any observed deviation from the standard PPE donning or doffing procedure that could potentially increase the risk of self-contamination, cross-contamination, or environmental contamination. After data collection, interception problems observed during the donning of PPE were classified into the several groups for data analysis. Interception problems during the process of donning PPE were divided into problems related to protective clothing, respirators, glove, and others (such as wrong position of the protective screen, uncovered hair, or exposed inner hat). Similarly, interception problems during the process of doffing PPE were divided into problems related to clothing, respirators, gloves, and other problems (including issues with undressing, inadequate hand hygiene, improper disposal of waste, and non-standard removal of the protective screen). “Looseness of the head and neck area” refers to the protective clothing is too loose, resulting in incomplete coverage of the head and neck skin, while “incorrect donning of the protective screen” indicates either incorrect positioning or failure to remove the protective film on the outside of the screen.

### Calculation formula

The proportion of the number of interception problems in donning and doffing PPE = the number of interception problems in donning and doffing PPE/person-times of donning and doffing PPE × 100%. The person-times are calculated by multiplying the number of individuals by the frequency of donning and doffing PPE.

### Statistical analysis

WPS 2019 software was used for statistical data, SPSS 23.0 for data analysis, and GraphPad 9.0 for plotting. The counting data were expressed by frequency and percentage, and the comparison between multiple groups was performed using the *X*^2^ test or Fisher exact test. A value of *P* < 0.05 was indicative of statistical significance.

## Results

### Proportion of the number of interception problems in donning and doffing PPE

The proportion of the number of interception problems in donning PPE was 5.29% (246/4,652), while during doffing PPE, it was 8.44% (382/4,525) (*P* < 0.001). Among cleaning, security, and maintenance personnel, the proportion of the number of interception problems during donning and doffing PPE was (11.21%, 11.58%, 21.21%) and (18.53%, 18.80%, 18.86%), respectively, significantly higher than that of medical personnel (all *P* < 0.001). Both doctors and cleaning personnel showed statistically significant differences in the proportion of interception problems during both donning and doffing of PPE: doctors 1.28% (7/545) in donning versus 4.78% (26/544) in doffing; cleaning personnel 11.21% (103/919) in donning versus 18.53% (166/896) in doffing (χ2 = 11.317 and 19.250, respectively; both *P* < 0.001)., as summarized in Table 1.

**Table 1:** Proportion of the number of interception problems in the process of donning and doffing PPE.

Personnel category	In the process of donning PPE			In the process of doffing PPE			Statistics	*P*
	Total person-times	Number of interception problems	Proportion of the number of problems (%)	Total person-times	Number of interception problems	Proportion of the number of problems (%)		
Doctor	545	7	1.28	544	26	4.78	11.317	< 0.001
Nurse	2,150	53	2.47	2190	67	3.06	1.425	0.233
Infection control personnel	467	1	0.21	252	2	0.79	1.323	0.282*
Cleaning personnel	919	103	11.21	896	166	18.53	19.250	< 0.001
Security personnel	406	47	11.58	468	88	18.80	8.694	0.003
Maintenance personnel	165	35	21.21	175	33	18.86	0.294	0.587
Total	4,652	246	5.29	4525	382	8.44	35.792	< 0.001
Statistics			255.553			318.065		
*P*			< 0.001			< 0.001		

*Using Fisher’s exact probability test.

### Distribution of interception problem types in donning PPE by personnel category

Among interception problems during donning PPE, protective clothing-related problems remained predominantly, followed by the problems related to respirators. Although the total number of interception problems varied among personnel categories (Table 1), the proportional distribution of the four problem types (protective clothing, respirator, glove, and other) was similar across personnel categories (*P* = 0.459), as shown in Table 2.

**Table 2:** Proportional distribution of problem types (protective clothing, respirator, glove, other) in donning PPE across personnel categories.

Personnel category	Total number of problems	Distribution of problems (%)				*χ²*	*P*
		Protective clothing problems	Respirator problems	Glove problems	Other problems		
Doctor	7	4 (57.143)	2 (28.571)	0 (0.000)	1 (14.286)	14.898	0.459*
Nurse	53	32 (60.377)	10 (18.868)	1 (1.887)	10 (18.868)		
Infection control personnel	1	1 (100.000)	0 (0.000)	0 (0.000)	0 (0.000)		
Cleaning personnel	103	79 (76.699)	16 (15.534)	1 (0.971)	7 (6.796)		
Security personnel	47	36 (76.596)	3 (6.383)	2 (4.255)	6 (12.766)		
Maintenance personnel	35	24 (68.571)	8 (22.857)	0 (0.000)	3 (8.571)		
Total	246	176 (71.545)	39 (15.854)	4 (1.626)	27 (10.976)		

*Using Fisher’s exact probability test.

### Distribution of interception problems in doffing PPE

Among interception problems in the process of doffing PPE, the major ones were protective clothing problems and other problems. Although the total number of interception problems varied among personnel categories (Table 1), the proportional distribution of the four problem types (protective clothing, respirator, glove, and other) was similar across personnel categories (*P* = 0.610), as shown in Table 3.

**Table 3:** Proportional distribution of problem types (protective clothing, respirator, glove, other) in doffing PPE across personnel categories.

Personnel category	Total number of problems	Distribution of problems (%)				χ²	*P*
		Protective clothing problems	Respirator problems	Glove problems	Other problems		
Doctor	26 (100)	11 (42.308)	1 (3.846)	0 (0.000)	14 (53.846)	12.895	0.610*
Nurse	67 (100)	37 (55.224)	4 (5.970)	4 (5.970)	22 (32.836)		
Infection control personnel	2 (100)	2 (100.000)	0 (0.000)	0 (0.000)	0 (0.000)		
Cleaning personnel	166 (100)	82 (49.398)	7 (4.217)	6 (3.614)	71 (42.771)		
Security personnel	88 (100)	43 (48.864)	7 (7.955)	1 (1.136)	37 (42.045)		
Maintenance personnel	33 (100)	21 (63.636)	0 (0.000)	1 (3.030)	11 (33.333)		
Total	382 (100)	196 (51.309)	19 (4.974)	12 (3.141)	155 (40.576)		

*Using Fisher’s exact probability test.

### Specific interception problems in donning and doffing PPE

**Fig. 1:**
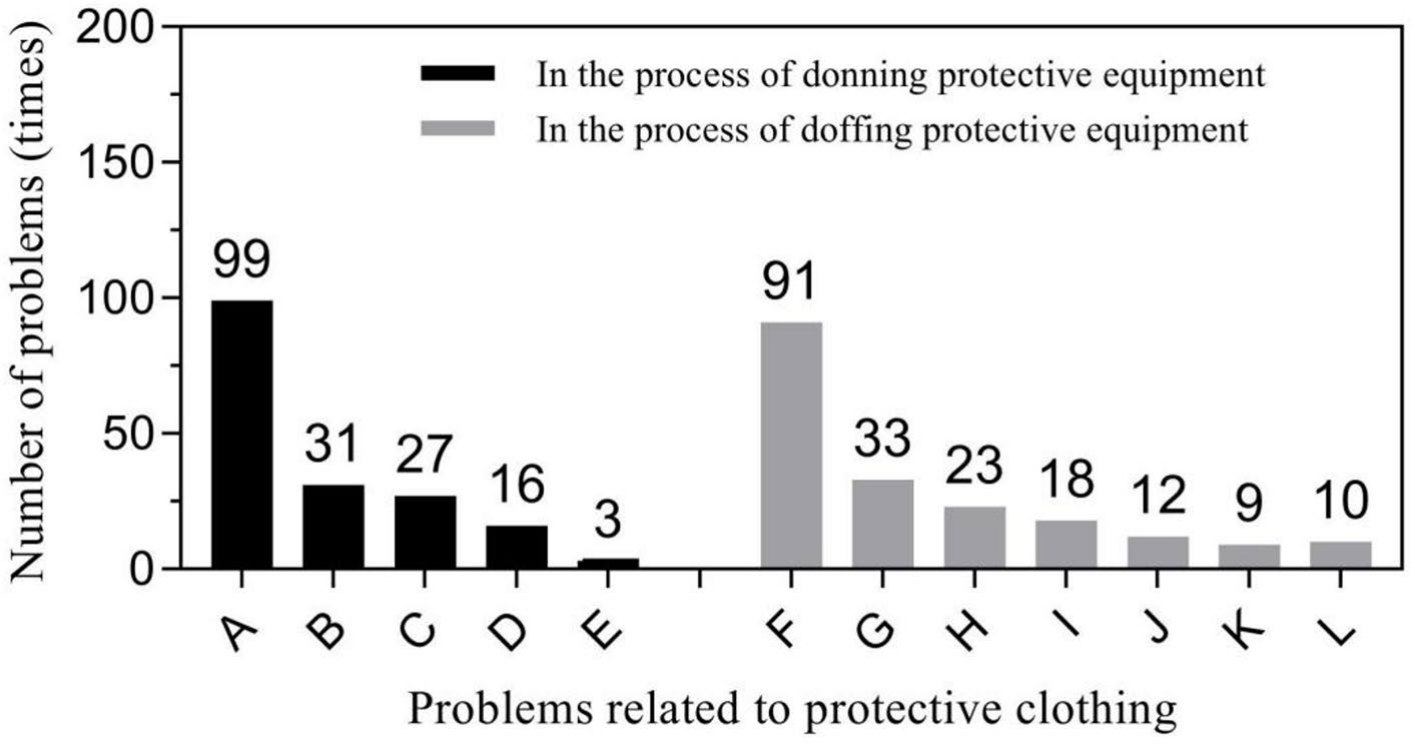
Problems related to protective clothing in the process of donning and doffing PPE. (1) in the process of donning PPE: A - the head and neck of the protective clothing were loose and easy to be exposed; B - the protective clothing covered the face or respirators; C - the protective clothing was damaged; D - the protective clothing model did not fit; E - other problems (zippers and seals were damaged). (2) In the process of doffing PPE: F - the inner surface of the protective clothing was contaminated; G - the inner clothing or socks were contaminated; H - incapable of doffing protective clothing or unfamiliar with the process of doffing protective clothing; I - the protective clothing was not rolled in or the protective clothing was rolled in irregularly; J - the protective clothing was removed before zipping to the end; K - the skin was contaminated; L - the inner cap or shoe cover was contaminated.

**Fig. 2:**
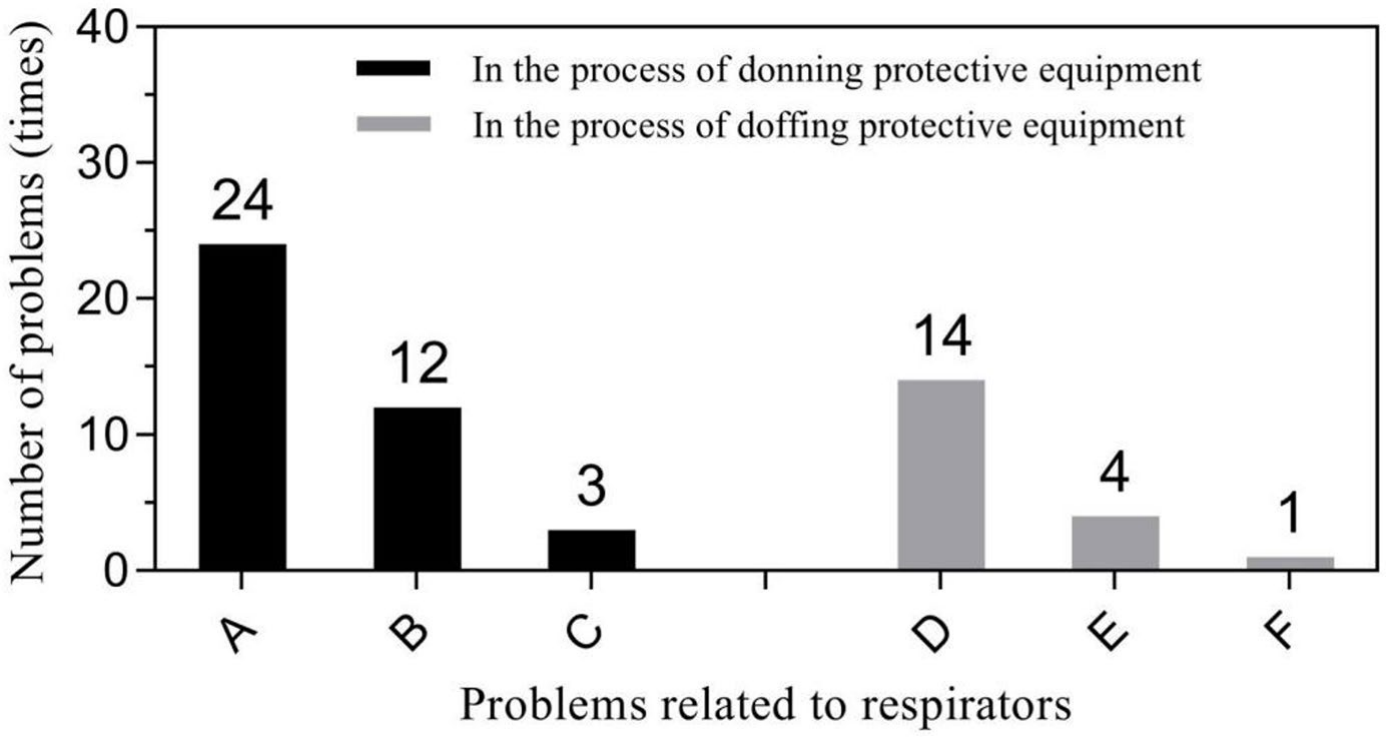
Problems related to respirators in the process of donning and doffing PPE. (1) in the process of donning PPE: A - the seal test was not passed, and the fluctuation was not obvious; B - the respirators was deformed or displaced; C - donning a double-layer respirators. (2) In the process of doffing PPE: D - the respirators was found to be displaced or loose when doffing; E - the respirators polluted the inner protective clothing; G - the respirators polluted the inner surface of the protective clothing.

**Fig. 3:**
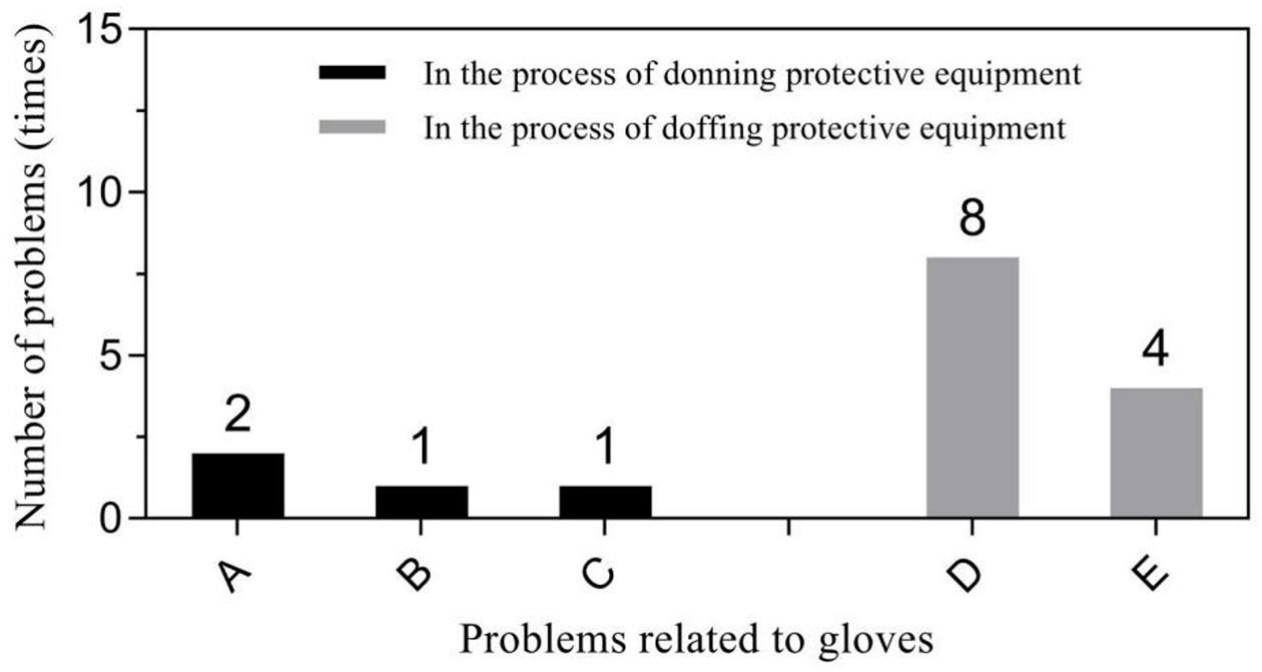
Problems related to gloves in the process of donning and doffing PPE. (1) in the process of donning PPE: A - the glove was damaged; B - the glove size was not suitable; C - the adhesive tape was wrapped too tightly. (2) In the process of doffing PPE: D - the inner glove was contaminated; E - the glove was damaged.

**Fig. 4:**
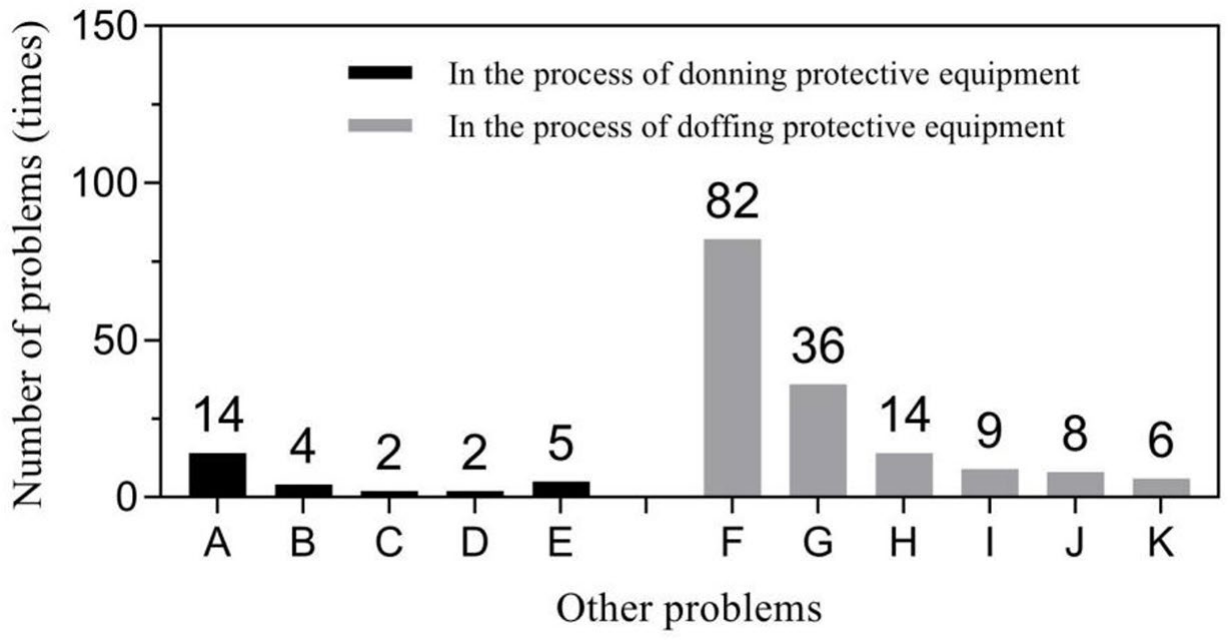
Other problems in the process of donning and doffing PPE. (1) in the process of donning PPE: A - problems of protective screen; B - inner cap exposure; C - a large area of wound on the skin; D - problems of glasses; E - other problems (chatting when changing the respirators, not donning shoes cover, etc.). (2) In the process of doffing PPE: F - non-standard hand hygiene; G - too large action when doffing and throwing off articles; H - inner cap or shoe cover falling off; I - personal belongings falling off; J - non-standard removal of protective screen; K - other problems (falling down, touching the mirror with the head, etc.).

Primary problems related to protective clothing included loose head and neck areas of the protective clothing leading to exposure during donning (56.25%, 99/176), and inner surface pollution of protective clothing during doffing (46.43%, 91/196). Respirator related problems mainly focused seal test failure during donning (61.54%, 24/39) and displacement or looseness of respirators during doffing (73.68%, 14/19). Glove-related problems predominantly included glove breakage (50%, 2/4) during donning and contamination of inner gloves during doffing (66.67%, 8/12). “Other” problems were diverse but were mainly concentrated in two specific behaviors: incorrect donning of the protective screen (51.85%, 14/27) and non-standard hand hygiene during the removal (52.90%, 82/155). Figures 1, 2, 3 and 4 show further details.

## Discussion

This study systematically evaluated the patterns of “interception problems” during PPE procedures in a large scale mobile cabin hospital, providing a comprehensive map of biosafety vulnerabilities. The primary highlight of our findings is the significant disparity in risk between the two procedures, with the doffing process exhibiting a substantially higher error rate (8.44%) compared to donning (5.29%). Furthermore, while non medical personnel (cleaning, security, and maintenance staff) were identified as the highest risk groups, the consistent distribution of error types, primarily centered on protective clothing and respirators, across all occupations suggests a universal pattern of technical difficulty in PPE usage. These findings are of significant clinical importance as they provide a data driven evidence base for administrators to transition from generalized training to “precision infection control,” focusing resources on high risk doffing steps and vulnerable staff categories to minimize occupational exposure.

The implementation of infection control supervisor system has proven effective in COVID-19 prevention and control efforts^24,25^, serving as a crucial mechanism for guiding and rectifying PPE donning and doffing procedures. In this study, the role of the infection control supervisor was further upgraded. Unlike previous part-time sensory control supervisors primarily active during the day, the supervisors in this study were full-time and available round-the-clock. Not only were full-time infection control personnel appointed, but also a dedicated infection control team was established for ongoing quality control. This ensured that issues documented during the donning and doffing of PPE were genuine and standardized, enhancing their analytical and reference value. This study found that during the process of donning PPE, there was a notably high incidence of interception problems among cleaning, security, and maintenance personnel, accounting for over 10%, with maintenance personnel reaching 21.21%. Similarly, the proportion of the number of interception problems during doffing PPE exceeded 18% among cleaning, security, and maintenance personnel, significantly higher than that observed among medical personnel. Previous relevant studies have also obtained consistent findings^13,26^.

Non-medical personnel, including cleaning, security, and maintenance personnel, have not previously undergone systematic training during donning and doffing PPE, which may contribute to their elevated infection rate. Given their essential role in the functioning of mobile cabin hospitals, prioritizing their training in PPE procedures is imperative for both personal safety and overall infection control effectiveness. Efforts should be made to assist them with an attitude of unwavering support and commitment. This could involve maintaining respectful communication, documenting daily issues, sharing them via WeChat groups for reminders, and providing targeted training videos to enhance their skills and awareness^27,28^.

Among the medical staff, nurses had the highest proportion of the number of interception problems during PPE donning (2.47%), whereas doctors exhibited the highest proportion during PPE doffing (4.78%). These findings are potentially attributed to the diverse origins of the medical teams, as ingrained habits from their original institutions may have conflicted with the standardized protocols. Regarding the distribution of interception problems, the problems related to protective clothing and respirators were predominant during PPE donning, while problems with protective clothing and other aspects were most prevalent during PPE doffing.

Regarding the distribution of specific problems during PPE donning, the issues were multifaceted, involving protective clothing, respirators, gloves, and other components. For protective clothing, problems mainly focused on the exposure of the head and neck, clothing damage, and improper sizing. For respirators, problems centered on seal test failures and respirator distortion or displacement, mirroring findings in relevant research on PPE assessment^21^. For gloves, issues primarily included glove breakage, improper sizing, and excessively tight tape winding. Other donning problems were concentrated on improper positioning of the protective screen and the exposure of the inner cap. These findings highlight the need to select appropriate models and sizes, inspect for damage before donning, and ensure material support provides high-quality protective equipment. To address these donning-related issues, we propose a ‘Targeted Donning Training Scenario.’ For example, (1) ‘Pre-Donning Inspection Drill’: staff are trained to systematically select appropriate sizes of protective clothing and gloves, and inspect for any damage before use; (2) ‘Mirror Self-Check Protocol’: after donning, staff must check for any exposed areas (head, neck, inner cap) in the mirror and make timely adjustments; (3) ‘Seal Test Reinforcement’: repeated practice of respirator seal tests until achieving consistent pass rates; and (4) ‘Tape-Winding Technique’: demonstration of appropriate tape tension to prevent glove breakage while maintaining secure attachment.

Doffing problems mainly involved inner surface contamination of protective clothing and contamination of inner garments or socks, consistent with findings in relevant literature^29^. Problems encountered during respirator removal primarily included respirator displacement or looseness and inner clothing contamination, indicating that some staff members in the cabin hospital may have experienced respirator displacement or looseness without noticing. Therefore, it is necessary to strengthen the inspection of the presence of PPE in the cabin hospital by infection control personnel. Other problems in doffing PPE were mainly the non-standard hand hygiene, the falling of the inner cap or shoe cover, the falling of personal belongings, and the non-standard removal of the protective screen. To mitigate these doffing-related risks, we recommend a ‘Simulation-Based Doffing Training Scenario.’ Specific examples include: (1) ‘Forward-Leaning Method’ training: staff practice removing respirators while leaning forward to prevent inner clothing contamination; (2) ‘Step-by-Step Hand Hygiene Drill’: repeated simulation of hand hygiene procedures between each doffing step to build muscle memory; (3) ‘Controlled Screen Removal’: practice removing protective screens by grasping the outer edges only, explicitly avoiding facial contact; (4) ‘No Personal Belongings Policy’: pre-entry reminders and pocket checks to prevent items from falling during doffing; and (5) ‘Buddy System Check’: pairing staff members to monitor each other’s doffing sequence for fallen inner caps or shoe covers. By combining these specific, evidence-based training scenarios with rigorous on-site inspection by infection control personnel, administrators can implement ‘precision infection control’ to effectively minimize occupational exposure.

This study was conducted in a large makeshift cabin hospital during the later stage of the COVID‑19 pandemic, when the outbreak had already been ongoing for more than two years. Although staff were still working under high workload and psychological stress, PPE and other essential supplies were generally adequate rather than severely limited. In this context, staff fatigue, anxiety, and heterogeneous professional backgrounds may have increased the likelihood of interception problems, while sufficient PPE availability and organized training may have partly mitigated these risks. Therefore, the frequency and pattern of interception problems observed in our study may not be fully generalizable to either the early, resource‑scarce phase of the pandemic or to routine hospital environments in non‑emergency periods.

This study has certain limitations. First, there is a risk of inadvertent self-contamination during the removal of PPE, emphasizing the importance of post-hand hygiene. However, there is a lack of quantification or guidance regarding the acceptable level of contamination during doffing PPE. Second, the lack of differentiation between specific problems among different job roles, which hinders the reflection of problem characteristics among personnel in varied positions. Third, the sample sizes for certain personnel categories, such as infection control staff and doctors, were relatively small compared to other groups. This disparity reflects the actual staffing structure of the mobile cabin hospital but may have limited the statistical power to detect significant differences in the distribution of interception problem types across all categories.

## Conclusions

In summary, the specific problems identified during PPE procedures in the mobile cabin hospital provide critical practical insights. These findings highlight the urgent need for targeted training programs designed to address these specific gaps. Prioritizing the rectification of these issues in future operations will be essential to minimize the risk of infection among healthcare personnel.

## Supplementary Information

Below is the link to the electronic supplementary material.

Supplementary Material 1

Supplementary Material 2

Supplementary Material 3

## Data Availability

Data is provided within the manuscript or supplementary information files.
